# Regulation and physiological functions of phoenixin

**DOI:** 10.3389/fmolb.2022.956500

**Published:** 2022-08-25

**Authors:** Han Liang, Qian Zhao, Shuangyu Lv, Xinying Ji

**Affiliations:** ^1^ The First Affiliated Hospital of Henan University, Henan University, Kaifeng, China; ^2^ Institute of Molecular Medicine, Henan International Joint Laboratory for Nuclear Protein Regulation, School of Basic Medical Sciences, Henan University, Kaifeng, China

**Keywords:** phoenixin, reproduction, inflammation, nesfatin-1, GPR173

## Abstract

Phoenixin is a newly discovered neuropeptide generated from small integral membrane protein 20. Phoenixin is a ligand for the G protein-coupled receptor 173 (GPR173) and has been detected in central and peripheral tissues of human, rats, mice, bovine, and zebrafish. It was initially involved in regulating reproductive function by stimulating the luteinizing hormone release from pituitary cells by increasing the level of gonadotropin-releasing hormone. Recently, many functions of phoenixin have been generalized, including regulation of food intake, memory, Alzheimer’s disease, anxiety, inflammation, neuronal and microglial activity, energy metabolism and body fluid balance, cardiovascular function, and endocrine activity. In addition, the interaction between phoenixin and nesfatin-1 have been revealed. The present article summarized the latest research progress on physiological function of phoenixin, suggesting that it is a potential target for novel drug development and clinical application.

## Introduction

**FIGURE 1:**
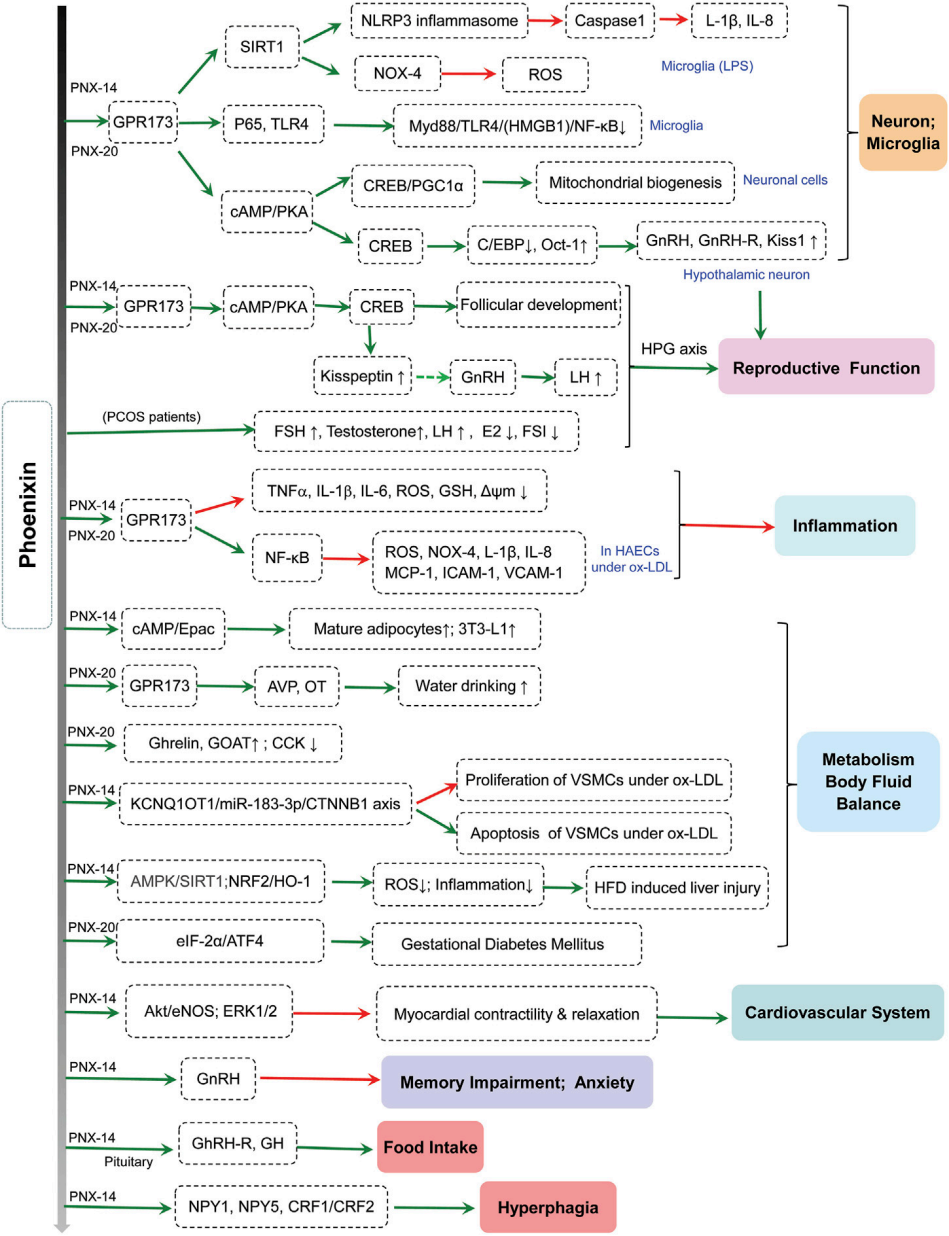
The physiological and pathological roles of phoenixin. AHA, Anterior hypothalamic area; Akt, protein kinase B; AMPK, AMP-activated protein kinase; ATF4, activating transcription factor 4; AVP, arginine vasopressin; cAMP, cyclic adenosine monophosphate; C/EBP, CCAAT/enhancer binding protein; CREB, cAMP-response element binding protein; CRF1/CRF2, corticotropin-releasing factor 1/2; CCK, cholecystokinin; E2, estradiol; eIF-2α, eukaryotic translation initiation factor 2α; eNOS, endothelial nitric oxide synthase; ERK, extracellular signal-related kinase; FSH, follicle-stimulating hormone; FSI, fasting serum insulin; GH, growth hormone; GhRH-R, growth hormone-releasing hormone receptor; GnRH, gonadotropin-releasing hormone; GnRH-R, gonadotropin-releasing hormone receptor; GOAT, ghrelin-O-acyl transferase; HAECs, human aortic endothelial cells; HFD, high-fat diet; HMGB1, high mobility group box1; HO-1, heme oxygenase-1; ICAM-1, intercellular adhesion molecule-1; IL-1β, interleukin-1β; IL-8, interleukin-8; LH, luteinizing hormone; LPS, lipopolysaccharide; MCP-1, monocyte chemotactic protein-1; Myd88, myeloid differentiation primary response 88; NF-κB, nuclear factor-κB; NLRP3, NOD-like receptor protein 3; NOX-4, NADPH oxidase 4; NPY1, neuropeptide Y1; NPY5, neuropeptide Y5; NRF2, Erythroid 2-related factor 2; NST, nucleus of the solitary tract; Oct-1, octamer-binding transcription factor-1; OT, oxytocin; ox-LDL, oxidized low-density lipoprotein; PCOS, polycystic ovary syndrome; PKA, protein kinase A; PGC-1α, peroxisome-proliferator-activated receptor coactivator (PGC)-1α; ROS, reactive oxygen species; SIRT1, Sirtuin1; TLR4, toll-like receptor 4; VCAM-1, vascular cell adhesion molecule-1; VSMCs, vascular smooth muscle cells; Δψm; mitochondrial membrane potential.

Phoenixin (PNX) is a novel neuropeptide that is cleaved from the C-terminal of small integral membrane protein 20 (SMIM20) (Lyu et al., 2013; Billert et al., 2020). The precursor for phoenixin is encoded by the gene *C4orf52* and synthesized on cytoplasmic ribosomes, and it is cleaved by ectodomain shedding, a regulated process whereby membrane proteins are cleaved (Hayashida et al., 2010; McIlwraith and Belsham, 2018). Phoenixin includes two active subtypes: phoenixin-14 (14 amino acids) and phoenixin-20 (20 amino acids). Phoenixin is a known potential ligand for GPR173, an isolated G-protein-coupled receptor (GPCR) (Stein et al., 2016; McIlwraith et al., 2019). Recent studies have reported that phoenixin could perform many functions through GPR173 (Billert, et al., 2020). Xuan et al. showed that phoenixin-20 accelerated the proliferation of granulosa cells (GCs), induced estradiol (E2) production, and promoted follicular growth through its receptor GPR173 (Xuan Phuoc et al., 2019). In addition, phoenixin-20 reduced lipopolysaccharide (LPS)-induced cytotoxicity by activating GPR173 (Sun et al., 2020). Recent studies have indicated that phoenixin is involved in various physiological and pathological functions (Figure 1).

## Expression of phoenixin

In rat brain, phoenixin and nesfatin-1 were co-localized to a large extent; hence, they are considered to have synergistic effects in some aspects. Presently, phoenixin has been detected in the central nervous system and peripheral tissues of rats, domestic pigs, spotted scat, zebrafish, chicken, and so on. Immunohistochemical results demonstrated that phoenixin immunoreactivity (irPNX) was found in the trigeminal spinal tract, superficial dorsal horn, and nucleus of the solitary tract (NTS), as well as in the cell population of dorsal root, trigeminal nerve, and nodular ganglion in the spinal cord of rats (Lyu, et al., 2013). Immunoreactive neural structures of the phoenixin protein in pig spinal cord showed that irPNX had a similar distribution pattern in grey matter of all spinal cord slices and varicose nerve fibers forming dense plexus, which could be observed mainly in the first and second layers of the dorsal horn (Lepiarczyk et al., 2020). Phoenixin-like and phoenixin receptor GPR173-like immunoreactivity was detected in zebrafish gonads and zebrafish liver (ZFL) cells (Rajeswari et al., 2022). In addition, phoenixin has been detected in the hypothalamus, pituitary gland, and liver. (Wang et al., 2018). Phoenixin is widely distributed in central and peripheral tissues, especially in the spinal cord and the hypothalamic-pituitary-gonadal (HPG) axis, which are closely related to neuroendocrine functions.

The intensity of phoenixin immunoreactivity in adult rat tissues from high to low was hypothalamus, heart, thymus, oesophagus, stomach, spleen, pancreas, lung, pituitary, and kidney (Yosten et al., 2013). In the hypothalamus, phoenixin was found in the arcuate nucleus (Arc), dorsal hypothalamus (DH), paraventricular nucleus (PVN), supraoptic nucleus (SON), ventromedial hypothalamus (VMH), and zona incerta (ZI) (Yosten, et al., 2013). Phoenixin was detected in magnocellular neurosecretory nuclei, and it suggested that phoenixin might be released into the circulatory system *via* the hypophyseal portal system (Palasz et al., 2015). In addition, phoenixin could be secreted by the peripherial tissues. Billert et al. reported that phoenixin was secreted from 3T3-L1 and rat primary adipocytes (Billert et al., 2018). Phoenixin was secreted from pancreatic islets, and the secretion of phoenixin depended on glucose concentration (Billert et al., 2019). Recent reports demonstrated that phoenixin is a multidimensional peptide (McIlwraith et al., 2021), indicating its roles in regulating many physiological and pathological functions (Table 1).

**TABLE 1:** Overview of the main function of phoenixin in recent literature.

Category	Species/strain/Cells	Treamrnt route	Form of phoenixin	Effect	References
Reproduction	Anterior pituitary cell from SD Rats	incubated, *in vitro*	PNX-14, PNX-20	GnRH receptor mRNA↑ LH ↑	Yosten, et al. (2013)
	SD Rats	ICV, acute	PNX-20	Plasma LH ↑	Stein et al. (2016)
	mHypoA	incubated, *in vitro*	PNX-20	GnRH and its mRA ↑	Treen, et al. (2016)
	HGrC1 cells	incubated, *in vitro*	PNX-14	Estradiol ↑, GCs ↑	Xuan Phuoc et al. (2019)
	Zebrafsh	IP, acute	PNX-20	mRNAs of GnRH, vitellogenin isoforms, and estrogen receptor ↑	Rajeswari et al. (2022)
	pituitary, spotted scat	incubated, *in vitro*	PNX-14, PNX-20	mRNAs of GnRHR, lh, fsh ↑	Wang et al. (2019)
	spotted scat	IP, acute	PNX-14, PNX-20	mRNAs of GnRHR, lh, fsh ↑	Wang et al. (2019)
	Wistar Rats	ICV, acute	PNX-14	Plasma FSH, LH, testosterone ↑	Guvenc, et al. (2019)
Food intake	SD Rats	ICV, acute	PNX-14	Light phase food intake ↑	Schalla, et al. (2017)
	spotted scat	Fasting	—	Hypothalamus phoenixin mRNA ↑	Wang et al. (2018)
	pituitary, spotted scat	incubated, *in vitro*	PNX-14, PNX-20	ghrhr, gh mRNA ↑	Wang et al. (2018)
	neonatal chickens	ICV, acute	PNX-14	Food intake ↑	Rajaei et al. (2022)
Memory and anxiety	KM mice	ICV, acute	PNX-14	Mitigate memory impairment induced by the Aβ1-42 and scopolamine	Jiang et al. (2015b)
	KM mice	ICV, acute	PNX-14	Anxiolytic effect	Jiang et al. (2015a)
		AHA injection		GnRH mRNA, plasma GnRH ↑	
Neuronal and microglial activity	SD rats with MCAO	ICV, acute	PNX-14	Protection against I/R-induced microglia	Ma et al. (2020)
	BV2 microglia cells	incubated, *in vitro*	PNX-14	Protection against OGD/R	Ma et al. (2020)
	BV2 microglial cells	incubated, *in vitro*	PNX-14	Ameliorate the activation of microglial NLRP3 inflammasome induced by LPS	Zeng et al. (2020)
	Brain slices of C57BL/6 mice	incubated, *in vitro*	PNX-14	Reduce the interictal-like event	Kalkan et al. (2021)
	Neuronal M17 cells	incubated, *in vitro*	PNX-20	Promotes neuronal mitochondrial biogenesis	Yang et al. (2020)
	Brain slices of SD Rats	incubated, *in vitro*	PNX-14, PNX-20	Influence the excitability of nucleus of the solitary tract neurons	Grover, et al. (2020)
	C57BL/6 mice with MCAO	ICV, acute	PNX-20	Ameliorates brain infarction by promoting microglia M2 polarization	Wang et al. (2022)
Inflammation	BV2 microglia cells	incubated, *in vitro*	PNX-14	TNFα, IL-1β, IL-6 ↓	Ma et al. (2020)
	Primary astrocytes from C57BL/6 mice	incubated, *in vitro*	PNX-14	LPS-induced infammation and infammasome activation ↓	Wang et al. (2020)
	Human dental pulp cells	incubated, *in vitro*	PNX-20	Anti-inflammatory effects	Sun et al. (2020)
	SD Rats	SC, acute	PNX-14	Alleviate indomethacin-induced duodenal ulcer, anti-inflammation	Zandeh-Rahimi et al. (2022)
Energy metabolism and body fluid balance	3T3-L1 fibroblast cell	incubated, *in vitro*	PNX-14	Promote preadipocytes differentiation	Billert et al. (2018)
	C57BL/6 mice with high fat diet	gastrogavage chronic	PNX-14	Inhibit non-alcoholic fatty liver disease, inhibit inflammation and ROS	Yang et al. (2020)
	Genetic GDM mouse model	IP, chronic	PNX-20	Ameliorates GDM symptom, placental oxidative stress, inflammatory cytokines, and ER stress.	Chi et al. (2021)
	SD Rats	ICV, acute	PNX-20	Water drinking ↑	Haddock et al. (2020)
	SD Rats	ICV, acute	PNX-20	Plasma vasopressin ↑	Gasparini et al. (2018)
	SD Rats	restraint stress	—	Brain nuclei phoenixin mRNA ↑	Friedrich et al. (2020)
	MGN3-1 cells, STC-1 cells	incubated, *in vitro*	PNX-20	Ghrelin, GOATs mRNAs ↑ CCK mRNA ↓	Mukherjee and Unniappan, (2021)

## Function of phoenixin

### Reproductive function

The hypothalamus, pituitary gland, and gonads produce neurohormones that are the vital regulatory hormones that promoting sexual maturity and maintain reproductive function. Recent literature suggested that phoenixin exerted roles in HPG axis activity and stress-induced fertility disorders (Yosten, et al., 2013; Golyszny et al., 2022). It was firstly found that phoenixin regulated pituitary gonadotropin secretion by modulating expression of the gonadotropin-releasing hormone (GnRH) receptor (Yosten, et al., 2013). Phoenixin/smim20 gene and protein are expressed in the brain including the hypothalamus regions important in the control of reproduction in adult zebrafish (Ceriani et al., 2021). Phoenixin has been proved to as an essential mediator in regulating ovarian periodicity. Phoenixin enhances the secretion of luteinizing hormone (LH) stimulated by GnRH. Moreover, it shows a direct effect on gonadotropins (Stein, et al., 2016). Phoenixin-20 regulates the expression of GnRH by reducing the expression of the *C/EBP* gene and increasing the expression of *Oct-1*. C/EBP and OCT-1 are two essential transcription factors of the GnRH promoter (Schalla and Stengel, 2018; Treen A. K. et al., 2016). Rybska et al. found that the development of canine uterine disorders, including endometrial hyperplasia or pyometra induced a downregulation of phoenixin and its receptor GPR173 expression (Rybska et al., 2022). Phoenixin can activate the cAMP/PKA pathway and lead to CREB phosphorylation through GPR173, and it induced an increase expression of genes related to follicular development (Xuan Phuoc, et al., 2019). Based on these results, phoenixin had quite a little expression in the hypothalamus in central nervous system, and it played a role in regulating HPG activity at multiple levels by activating GPR173 and GnRH receptor.

Recently, it was showed that phoenixin had a role in regulating HPG hormones and reproductive processes in fish (zebrafish and spotted scat). Phoenixin-like and putative phoenixin receptor Gpr173-like immunoreactivity was detected in zebrafish gonads and ZFL cells (Rajeswari and Unniappan, 2020). The genes involved in the steroid production pathway (*cyp11a1*, *cyp17a1*, *17βhsd*, and *cyp19a1a*) were upregulated in the gonads of fish administered with phoenixin-20, and the expression of vitellogenin isoforms and estrogen receptor (*esr2a* and *esr2b*) mRNAs were upregulated in ZFL cells *in vitro*. Phoenixin-20 stimulated mRNAs encoding HPG hormones, such as regulation of vitellogenin and upregulation of steroidogenic mRNAs in the gonads (Rajeswari and Unniappan, 2020). In spotted scat, phoenixin can stimulate reproduction not only through the HPG axis but also directly through the pituitary gland (Wang et al., 2019). It is noteworthy that the phoenixin protein is dominant in the female reproductive system, because the stimulating effect of this protein on the HPG axis could be detected only in female cells or animals. However, even in male rats, phoenixin exerts a certain influence on the regulation of male sex hormones, which may not be through the activation of the HPG axis.

Phoenixin and nesfatin-1 have a similar distribution pattern in the brain. Both phoenixin and nesfatin-1 significantly increased the secretion of follicle-stimulating hormone (FSH), LH, and testosterone in rat plasma without inducing any changes in plasma GnRH, thus indicating that both these neuropeptides play a synergistic role in regulating male sex hormones (Guvenc et al., 2019). Nesfatin-1, a polypeptide containing 82-amino acid, was derived from the precursor protein nucleobindin 2, and it was secreted by the hypothalamus (Oh et al., 2006). Nesfatin-1 had a widespread distribution in central and peripheral tissues, and it was implicated in multiple physiological and pathological processes, including glucose and lipid metabolism, gastrointestinal functions, thermogenesis, depression, anxiety, as well as cardiovascular and reproductive functions (Luo et al., 2021).

A clinical study showed that the concentrations of LH, FSH, and nesfatin-1 in patients with PCOS were positively correlated with the level of phoenixin (Ullah et al., 2017). The nesfatin-1 play a dual effect in reproductive physiology. The nesfatin-1 level was lower in polycystic ovary syndrome (PCOS), and the decrease of nesfatin-1 may contribute to the mechanism governing PCOS, according the analysis of patients with PCOS (Demir Çaltekin et al., 2021) and animal model with PCOS (Xu et al., 2017). However, Ademoglu et al. reported that it had high concentrations of serum nesfatin-1 in PCOS patients (Ademoglu et al., 2014). The serum insulin in patients with PCOS was significantly higher than control group (Ullah, et al., 2017). In contrast, the concentrations of E2 and fasting serum insulin (FSI) were significantly negatively correlated with the level of phoenixin (Ullah, et al., 2017; Kalamon et al., 2020). These results indicate that phoenixin may play a role in developing of PCOS. Kulinska et al. demonstrated that the reduced phoenixin serum level and GPR173 expression might contribute to HPG axis dysregulation in women with endometriosis (Kulinska et al., 2021). In patients with endometriosis, the phoenixin and LH/FSH ratio had diagnostic relevance (Kulinska, et al., 2021), suggesting a significance of phoenixin in the diagnosis and treatment of endometriosis. More clinical research is wanted for an application of phoenixin in endometriosis and related diseases.

### Food intake

Phoenixin immunoreactivity was detected in the Arc and NTS (Yosten, et al., 2013; Prinz and Stengel, 2017), and its receptor GPR173 was expressed in the Arc and PVN (Stein, et al., 2016; Treen Alice K. et al., 2016). These brain areas are closely related to the regulation of food intake (Prinz et al., 2017; Schalla and Stengel, 2019), thus suggesting a potential role of phoenixin in food intake. The phoenixin expression in the hypothalamus was modulated by peripheral signals, such as hormones and fatty acids (McLlwraith et al., 2018). It was speculated that diet could stimulate phoenixin secretion, act on GnRH neurons and induce subsequent activation of GnRH (Valsamakis et al., 2021). It was reported that an intracerebroventricular (i.c.v.) injection of phoenixin-14 under illumination induced a dose-dependent increase in food intake in rats. Specifically, the in-depth evaluation of food intake showed that i.c.v. injection of phoenixin-14 induced an increase in meal size, meal duration, meal time, and food intake rate, while the interval between meals and the satiety ratio decreased (Schalla et al., 2017). However, when phoenixin-14 was i.c.v. injected in dark or injected intraperitoneally under light, it did not affect food intake (Schalla, et al., 2017). In addition, phoenixin was involved in regulating the feeding of spotted scat. After fasting for 2 days and 7 days, the expression of the phoenixin protein in the hypothalamus was increased significantly, but decreased significantly after feeding again, and the mRNA level of ghrhr (growth hormone-releasing hormone receptor) and gh (growth hormone) was up-regulated in the pituitary after treatment with phoenixin-14 *in vitro* (Wang, et al., 2018). Therefore, it was speculated that phoenixin might be involved in regulating the feeding of spotted scat by increasing the expression of ghrhr and gh in the pituitary gland (Wang, et al., 2018). In neonatal chickens, i.c.v. administration of phoenixin-14 induced hyperphagia, which was mediated by neuropeptide Y1 (NPY1), neuropeptide Y5 (NPY5), and corticotropin-releasing factor 1/2 (CRF1/CRF2) receptors (Rajaei et al., 2022). In patients with anorexia nervosa (AN), the serum phoenixin level was decreased in malnourished patients (accAN) (Palasz et al., 2019). Phoenixin may play an important role in the etiology and course of AN. Phoenixin level is closely related to some eating disorder symptoms, thus indicating its potential role in regulating food intake in patients with AN (Palasz, et al., 2019).

### Memory and Alzheimer’s diseases

Neuropeptides are commonly involved in regulating learning and memory. Phoenixin-14 (i.c.v.) promotes memory formation and prolongs memory retention time for tasks of novel object recognition and target location recognition in animals. Phoenixin-14 injected into the hippocampus can also enhance memory, while its i.c.v. administration could alleviate memory impairment caused by amyloid-β1-42 peptide aggregation and scopolamine, and the GnRH receptor was involved in this process (Jiang et al., 2015b). Recently, a clinical study compared the levels of phoenixin in individuals with subjective memory impairment, mild cognitive impairment, and mild Alzheimer’s disease, and demonstrated that the average plasma phoenixin level in the mild cognitive impairment group was negatively correlated with logical memory (Yuruyen et al., 2017). The average plasma phoenixin level in the subjective memory impairment group was positively correlated with immediate recall; this finding suggested that the plasma phoenixin level may have a predictive effect on cognitive memory impairment (Yuruyen, et al., 2017). Phoenixin may be a potential target to enhance memory and treat Alzheimer’s disease.

### Anxiety

Phoenixin regulates the secretion of pituitary gonadotropins by enhancing the expression of the GnRH receptor gene (Millar and Newton, 2013; Yosten, et al., 2013). GnRH plays a role in regulating brain response to anxiety (Umathe et al., 2008; Telegdy et al., 2009), thus indicating a potential role of phoenixin in regulating anxiety. Phoenixin-14 exerted a dose-dependent anti-anxiety effect on mice in the open field test and elevated plus maze test, and the anti-anxiety effect appeared after the injection of phoenixin-14 into the anterior hypothalamus (AHA), not in the amygdala (Jiang et al., 2015a). It is assumed that phoenixin might be involved in the mechanism of anxiety regulation (Palasz et al., 2018). In clinical trials, after evaluating depression (PHQ-9) and perceived stress (PSQ-20) of 68 inpatients with extensive psychometric anxiety (GAD-7), it was found that the phoenixin level was negatively correlated with anxiety in obese men (Hofmann et al., 2017). A recent study indicated that restraint stress reduced circulating phoenixin levels in male rats (Schalla et al., 2020). Thus, phoenixin plays a crucial role in emtional behavior, such as anxiety. However, whether phoenixin had an effect on neurotransmitter or nervous excitation need to be investigated.

### Neuronal and microglial activity

Phoenixin was shown to stimulate the excitability of nerve cells and promote the occurrence of mitochondrial organisms. Phoenixin-20 promoted the mitochondrial biogenesis of neurons mainly by regulating the CREB/PGC-1α pathway. Specifically, the expression levels of mitochondrial regulatory factors, including PGC-1α, NRF-1, and TFAM, were increased in cultured neuronal M17 cells after treatment with phoenixin-20 (Yang, et al., 2020). In mHypoA cell lines, phoenixin-20 can stimulate the expression of the *GnRH*, *GnRH-R*, and *Kiss1* genes through GPR173 by activating the cAMP/PKA pathway of CREB or through the expression of *C/EBP* and/or *Oct-1* (Treen, et al., 2016). In microglia, phoenixin-14 inhibited the signaling pathway of myeloid differentiation primary response 88 (Myd88)/toll-like receptor 4 (TLR4)/high mobility group box 1 (HMG B1)/nuclear factor-κB (NF-κB) by preventing the nuclear translocation of p65 protein and decreasing the activation of TLR4 (Ma et al., 2020). In BV2 microglia, phoenixin-20 exhibits an inhibitory effect on NOD-like receptor protein 3 (NLRP3) inflammasome induced by LPS, subsequently leading to the suppression of IL-1β and IL-18 secretion; moreover, the neuroprotective effect of phoenixin-20 was dependent on silent information regulator 1 (Zeng et al., 2020). In addition, phoenixin-20 significantly inhibits the NADPH oxidase 4 (NOX-4) and reactive oxygen species (ROS) induced by LPS (Zeng, et al., 2020). Electrophysiological recordings from brain slices demonstrated that phoenixin-14 reduced the interictal-like events in the entorhinal cortex and hippocampus (Kalkan et al., 2021), suggesting a potential role of phoenixin-14 in epileptiform activity. Recent study demonstrated that inflammatory stress induced by LPS up-regulated phoenixin expression and activity in the specific brain nuclei of SD Rats, indicating that several stressors could activate phoenixin signaling (Friedrich et al., 2022).

Phoenixin-20 also increased the ratio of mitochondrial DNA to nuclear DNA (mtDNA/nDNA) and promoted the expression of multiple mitochondrial genes and the mitochondrial respiratory rate and ATP production (Yang, et al., 2020). The role of phoenixin-20 in neuronal cells suggests that it could be considered as a therapeutic target for neurodegenerative diseases (Yang, et al., 2020). Phoenixin-20 can also directly control the excitability of nucleus of NTS neurons. Phoenixin increased the discharge frequency of some NTS neurons, and nearly 50% of NTS neurons were depolarized after treatment with phoenixin-20 (Grover et al., 2020). Wang et al. found that phoenixin-20 could mitigate brain infarction by promoting microglia M2 polarization using an ischemic stroke middle cerebral artery occlusion (MCAO) mouse model, and it was dependent on GPR173 (Wang et al., 2022). *In vitro* experiments demonstrated that phoenixin-14 alleviated morphine-induced cellular senescence in M17 neuronal cell through regulating yes-associated protein expression (Hu et al., 2022). In brief, phoenixin exerted a role on neuroprotection mainly by suppressing inflammation and ROS, and the experiments using human neuron and glial cells *in vitro*, and clinical studies are wanted to promote its applied research.

### Regulation of inflammation

Neuroinflammation commonly occurs in the aging brain and several diseases, such as Alzheimer’s disease, Parkinson’s disease, Acquired Immune Deficiency Syndrome dementia complex, and stroke (Vesce et al., 2007). LPS induces inflammation of astrocytes and activates inflammatory bodies, and it inhibits the gene and protein expression of GPR173 (Wang et al., 2020). Phoenixin-14 can attenuate the production of ROS and decrease the level of superoxide dismutase (SOD) induced by LPS (Wang, et al., 2020). Phoenixin-14 can regulate the expression of proinflammatory cytokines, such as tumor necrosis factor-α (TNFα), interleukin-1β (IL-1β), and interleukin-6 (IL-6), and it can reduce the generation of ROS and increase the level of glutathione induced by oxygen-glucose deprivation/reperfusion injury in BV2 microglia (Ma, et al., 2020). Phoenixin-14 was found to increase the expression of protective nitric oxide synthase and nitric oxide, and decrease the permeability of the endothelial monolayer in human brain endothelial bEnd.3 cells (Zhang and Li, 2020).

Phoenixin-20 inhibited LPS-induced inflammation of dental pulp cells, and phoenixin-20 inhibited the release of pro-inflammatory cytokines and inflammatory mediators induced by LPS, including monocyte chemotactic protein-1 (MCP-1), IL-6, intercellular adhesion molecule-1 (ICAM-1), vascular cell adhesion molecule-1 (VCAM-1), MMP-2 and MMP-9 (Sun, et al., 2020). Phoenixin-20, activating GPR173, significantly ameliorated oxidized low-density lipoprotein (ox-LDL) induced harmful effects by suppressing the NF-κB pathway in human aortic endothelial cells, and the harmful effects were presented as an increase in ROS, NOX-4, IL-1β, interleukin-8 (IL-8), and MCP-1 expression, as well as ICAM-1 and VCAM-1 release (Wei et al., 2021). Recently, Zandeh-Rahimi et al. found that phoenixin-14 could protect indomethacin-induced duodenal ulcer in rats, and phoenixin-14 exhibited a decrease in serum levels of inflammatory cytokines (IL-1β, TNF-α, IL-6, and IL-12), malondialdehyde and myeloperoxidase activity, and an increase on SOD and catalase activity in duodenal ulcer (Zandeh-Rahimi et al., 2022). This result indicated that phoenixin-14 alleviated duodenal ulcer against inflammation *via* reducing inflammatory cytokines and suppressing oxidative content. On the basis of these findings, phoenixin might be used as a new potential therapeutic agent for ischemic stroke, gastrointestinal inflammatory disorders, and other central nervous system disorders.

### Energy metabolism and body fluid balance

Phoenixin is positively correlated with human body mass index, indicating it may play a potential role in regulating body weight (Billert, et al., 2018). The *Smim20* and *Gpr173* genes were detected in 3T3-L1 and rat primary preadipocytes, and phoenixin-14 stimulated the transformation of 3T3-L1 and rat primary preadipocytes into mature adipocytes through the cAMP/Epac-dependent pathway (Billert, et al., 2018). Phoenixin could promote the production of white adipocytes, and it may be involved in body weight control (Billert, et al., 2018). Phoenixin-14 alleviated high-fat diet-induced liver injury through activation of the AMP-activated protein kinase/Sirtuin1 and Erythroid 2-related factor 2/heme oxygenase-1 (NRF2/HO-1) pathways in experimental nonalcoholic fatty liver disease mice (Yang, et al., 2020). Recently, Chi et al. found that phoenixin-20 ameliorated gestational diabetes mellitus (GDM) symptoms in the GDM mouse model, and phoenixin-20 could significantly inhibit the activation of eukaryotic translation initiation factor 2α/activating transcription factor 4 endoplasmic reticulum (ER) stress signaling pathway in GDM mice (Chi et al., 2021). In streptozotocin-induced diabetes mice, phoenixin-14 protected against cardiac damages through the sirtuin-3 pathway (Yao et al., 2021). In diabetes mouse model, phoenixin could alleviate symptom or protect against cardiac damage, and the related factors, gene, and ER were involved in these processes.

Intraventricular administration of phoenixin stimulated drinking in rats under random conditions and increased the amount of drinking water under thirst at night. In addition, pretreatment with losartan blocked the effect of phoenixin (Haddock et al., 2020). Both phoenixin and its receptor GPR173 are expressed in the SON and PVN of the hypothalamus, and phoenixin may affect the secretion of arginine vasopressin or oxytocin (Gasparini et al., 2018). In zebrafish, phoenixin-20 exerted as a tissue-specific regulator of the liver (suppressor) and muscle (stimulant) insulin-like growth factor signaling (Rajeswari et al., 2022). Phoenixin may be as a regulator for the homeostasis of body fluids and electrolytes, which due to its expressions areas in SON and PVN in hypothalamus.

Exogenous phoenixin induced a reduction of contractility and relaxation of isolated Langendorff-perfused rat hearts, without affecting coronary artery pressure and heart rate, and these effects were accompanied by the increase in phosphorylation of Erk1/2, Akt, and eNOS; these changes may be the reason for alteration in cardiac contractility and diastole function (Rocca et al., 2018). Phoenixin-14 inhibited cell proliferation and facilitated apoptosis of vascular smooth muscle cells under ox-LDL treatment by regulating the KCNQ1OT1/miR-183-3p/CTNNB1 axis (Ling et al., 2021). In addition, phoenixin treatment of isolated hearts with ischemia/reperfusion (I/R) injury showed a smaller infarct area and better contraction recovery than the control group (Rocca, et al., 2018). This demonstrated that phoenixin plays a protective role in the presence of I/R.

### Endocrine regulation

Phoenixin expression is regulated by bisphenol A (BPA), fatty acid palmitate, docosahexaenoic acid (DHA), and oleate (McLlwraith, et al., 2018). The BPA, as an endocrine disrupting chemical, has estrogenic activity (Acconcia et al., 2015). The oleate and palmitoleate are monounsaturated fatty acids, and DHA is polyunsaturated fatty acid. These results demonstrated that phoenixin was responsive to estrogenic activity as well as multiple fatty acids. After treatment of immortalized hypothalamic neurons with palmitic acid or BPA for 2-24 h, the expression level of the *Gpr173* gene was decreased (McLlwraith, et al., 2018). Furthermore, pretreatment with palmitic acid blocked phoenixin-induced phosphorylated cAMP response element-binding protein (CREB) level (McIlwraith, et al., 2019). GnRH-R analogues (bacarelin and cetrorelix) play a role in phoenixin/smim20 signal transduction of the HPG axis (Suszka-Switek et al., 2019). The expression level of the *mitomycin-20* gene was increased in the pituitary gland and especially in the ovary after the administration of bacarelin and cetrorelix in female rats (Suszka-Switek, et al., 2019). GnRH analogues may act as a factor in regulating reproductive function by affecting the expression of phoenixin. In addition, physical effects and restraint stress may affect the expression of phoenixin. Restraint stress significantly increased the expression of phoenixin in many brain nuclei (Friedrich et al., 2020). A recent study demonstrated that phoenixin-20 could act directly on gut cells to regulate metabolic hormones (Mukherjee and Unniappan, 2021). Phoenixin-20 significantly upregulated mRNA levels of *ghrelin* and ghrelin-O-acyl transferase (*GOAT*) in MGN3-1 (mouse stomach endocrine) cells, and markedly suppressed mRNA level of cholecystokinin (*CCK*) in STC-1 (mouse enteroendocrine) cells (Mukherjee and Unniappan, 2021). Celik et al. had demonstrated that phoenixin as well as endocan were higher in both aqueous humor and blood samples compared with the group of patients without diabetic retinopathy with cataract (Celik and Aydin, 2022), indicating a possible close relationship between phoenixin and diabetes mellitus.

## Interaction with nesfatin-1

In rat brain, nesfatin-1 showed a high degree of co-localization with phoenixin. Nearly one-fifth of cells in the PVN, Arc, ventromedial nucleus and lateral hypothalamic nucleus showed irPNX (Lyu, et al., 2013; Lyu et al., 2018). Bioinformatic survey demonstrated that both phoenixin and nesfatin were as ancient neuropeptides with premetazoan origin (Yanez-Guerra et al., 2022). The expression of nesfatin-1 in 50% of all neurons occurred at the same site, especially in the hypothalamic nucleus in rats, and most of the irPNX neurons showed co-expression with nesfatin-1 (Palasz, et al., 2015). Consequently, both phoenixin and nesfatin-1 were involved in regulating the neuroendocrine activity.

Both phoenixin and nesfatin-1 could significantly enhance the levels of FSH, LH, and testosterone in rat plasma without causing any changes in plasma GnRH level (Guvenc, et al., 2019). Phoenixin and nesfatin-1 can synergistically affect the plasma androgen level (Palasz, et al., 2015; Guvenc, et al., 2019). In addition, phoenixin can directly activate nesfatin-1 immunoreactive neurons in rat brain. After i.c.v. administration of phoenixin, the number of nesfatin-1 immunoreactive neurons was increased in the lateral septal nucleus, PVN, NTS, and SON (Friedrich et al., 2019). Recently, Friedrich et al. proposed that phoenixin had possible physiological interactions with nesfatin-1, especially in stress response (Friedrich and Stengel, 2021). Under restraint stress, the nesfatin-1 level in rat serum was increased, whereas the phoenixin level was decreased (Schalla, et al., 2020). In rats under restraint stress, both nesfatin-1 and phoenixin immunoreactivity was upregulated in the nucleus of the solitary tract (NTS) and raphe pallidus, however they exhibited different expression in other nuclei (Goebel et al., 2009; Friedrich, et al., 2020). The co-localization of phoenixin and nesftin-1 in several nucleus in hypothalamus suggested that the two homologous protein may play similar roles in neuroendocrine regulation. Recent reports demonstrated that phoenixin and nesftin-1 exhibited a same trend on HPG axis activity. In the circumstance of restraint stress, the phoenixin and nesftin-1 immunoreactivity areas in brain are different. The definite interaction and mechanism of phoenixin and nesftin-1 need to be furtherly investigated.

## Conclusion

Phoenixin, a recently discovered neuropeptide, plays a role in many physiological processes. In the past few years, our understanding of the role of phoenixin has dramatically improved, which is far beyond its originally described role in reproductive function. Phoenixin also regulates animal food intake, learning and memory, anxiety, inflammatory response, cardiac protection, body weight, adipocyte growth, and body fluid and electrolyte balance. Phoenixin plays a variety of roles by activating GPR173. Phoenixin mainly produces beneficial effects by inhibiting inflammation and oxidative stress *in vitro* or *in vivo* experimental disease models. Hence, it might be a promising target for the development of anti-inflammatory and antitumor drugs. Considering the physiological roles of phoenixin, its effects on endocrine disorders and neurodegenerative diseases remains to be further explored. In the future, gene knock-in/knock-out animal models and clinical studies are needed to clarify the physiological function of phoenixin and evaluate its therapeutic potential.

## Abbreviations

↑, increase; ↓, decrease; AHA, anterior hypothalamic area; fsh, follicular stimulating hormone; GCs, Granulosa cells; GDM, gestational diabetes mellitus; gh, growth hormone; GnRHR, gonadotropin releasing hormone receptor; I/R, ischemia/reperfusion; ICV, intracerebroventricular; IL-1β, interleukin-1β; IL-6, interleukin-6; IP, intraperitoneal; lh, luteinizing hormone; LPS, lipopolysaccharide; MCAO, middle cerebral artery occlusion; NLRP3, NOD-like receptor protein 3; OGD/R, oxygen-glucose deprivation and reoxygenation; ROS, reactive oxygen species; SC, subcutaneous; SD, sprague-dawley; TNFα, tumor necrosis factor α.
